# Identification of ABCC2 as a binding protein of Cry1Ac on brush border membrane vesicles from *Helicoverpa armigera* by an improved pull‐down assay

**DOI:** 10.1002/mbo3.360

**Published:** 2016-04-01

**Authors:** Zishan Zhou, Zeyu Wang, Yuxiao Liu, Gemei Liang, Changlong Shu, Fuping Song, Xueping Zhou, Alejandra Bravo, Mario Soberón, Jie Zhang

**Affiliations:** ^1^ State Key Laboratory of Rice Biology, Institute of BiotechnologyZhejiang UniversityNo. 866 Yuhangtang RoadXihu District, Hangzhou310012China; ^2^ State Key Laboratory for Biology of Plant Diseases and Insect Pests, Institute of Plant ProtectionChinese Academy of Agricultural SciencesNo. 2 Yuanmingyuan West RoadHaidian District, Beijing100193China; ^3^ Instituto de BiotecnologíaUniversidad Nacional Autónoma de MéxicoCuernavacaApdo. Postal 510‐3Morelos62250Mexico

**Keywords:** ABCC2, alkaline phosphatase, aminopeptidase‐N, *Bacillus thuringiensis*, cadherin, Cry1Ac, *Helicoverpa armigera*.

## Abstract

Cry1Ac toxin‐binding proteins from *Helicoverpa armigera* brush border membrane vesicles were identified by an improved pull‐down method that involves coupling Cry1Ac to CNBr agarose combined with liquid chromatography–tandem mass spectrometry (LC‐MS/MS). According to the LC‐MS/MS results, Cry1Ac toxin could bind to six classes of aminopeptidase‐N, alkaline phosphatase, cadherin‐like protein, ATP‐binding cassette transporter subfamily C protein (ABCC2), actin, ATPase, polycalin, and some other proteins not previously characterized as Cry toxin‐binding molecules such as dipeptidyl peptidase or carboxyl/choline esterase and some serine proteases. This is the first report that suggests the direct binding of Cry1Ac toxin to ABCC2 in *H. armigera*.

## Introduction

*Bacillus thuringiensis* is one of the most widely used biopesticides because of its specific insecticidal activity against target insects and its safety for humans and for the environment (Ibrahim et al. 2010; Pardo‐Lopez et al. 2013). Most of the Cry toxins are produced during the sporulation phase of bacterial growth (Bravo et al. 2007). In a complex multistep process including solubilization, activation, binding to different insect gut proteins, membrane insertion, and pore formation, Cry toxins kill the insects by disrupting midgut cells (Bravo et al. 2004; Pigott and Ellar 2007; Pacheco et al. 2009).

*Helicoverpa armigera* is a serious agricultural pest globally, it is one of the most damaging cotton pests in China and increased frequency of resistance to Cry1Ac cotton has been reported from field populations in northern China (Zhang et al. 2011). Thus, actions to understand the mechanism of action and resistance mechanism of Cry1Ac toxin in this pest are necessary.

Some proteins from insect midgut cells have been shown to bind Cry1A toxins, including cadherin (CAD) (Vadlamudi et al. 1993; Hua et al. 2014), aminopeptidase‐N (APN) (Nakanishi et al. 2002; Rajagopal et al. 2003; Bravo et al. 2004; Chen et al. 2009), alkaline phosphatase (ALP) (Jurat‐Fuentes and Adang 2004; Fernandez et al. 2006; Soberón et al. 2009; Arenas et al. 2010; Upadhyay and Singh 2011; Zuniga‐Navarrete et al. 2013), and actin among others (McNall and Adang 2003; Krishnamoorthy et al. 2007; Bayyareddy et al. 2009). Different strategies were used to identify these proteins as Cry1A‐binding proteins, for instance, several APN molecules have been shown to bind with different Cry toxins by using pull‐down Cry1A‐affinity chromatography assays followed by elution with different buffers: APN1 from *Heliothis virescens* was eluted with *N*‐acetyl‐galactosamine (GalNAc) containing buffer as Cry1Ac binding to APN1 relies on GalNac moieties (Luo et al. 1997); in the case of APN2 from *Manduca sexta*, it was eluted using a high‐pH carbonate buffer (Denolf et al. 1997), while APN4 from *H. virescens* was eluted with 2.0 mol/L NaSCN buffer (Banks et al. 2001). In this study, we used an improved pull‐down method that in contrast with the previously reported pull‐down assays does not require the elution step. Liquid chromatography–tandem mass spectrometry (LC‐MS/MS) was used for the identification of native proteins from microvilli membrane of midgut cells isolated from *H. armigera* that bind to Cry1Ac trypsin‐activated toxin. Here we show that the method used to pull down Cry1Ac‐binding proteins allowed the identification of a wide range of insect gut proteins. Some of these proteins had been previously identified by the different methods reported earlier. However, the pull‐down strategy presented here identified several other molecules that had not been characterized as Cry1Ac‐binding molecules. Among these, an ABCC2 transporter, previously showed to be involved in Cry1A action since resistance in different lepidopteran species was linked to ABCC2 mutations (Gahan et al. 2010; Baxter et al. 2011; Atsumi et al. 2012; Xiao et al. 2014), was identified as a Cry1Ac‐binding protein. The role of ABCC2 transporter in the mechanism of action of Cry1A toxin is not known, but it has been proposed that it is a receptor protein involved in facilitating insertion of Cry1A oligomers into the membrane (Heckel 2012). Nevertheless, a direct interaction of Cry1A with ABCC2 from insect gut was not demonstrated until now. Revealing the role of ABCC2 in Cry1Ac mode of action is important for the future improvement of these toxins and for developing tools to counter insect resistance.

## Experimental Procedures

### Production of Cry1Ac toxin

The HD73 strain producing Cry1Ac toxin was grown in 1/2 LB medium (0.5% NaCl, 0.5% tryptone, and 0.25% yeast extract, w/v) at 220 rpm and 30°C as described previously (Zhou et al. 2015). Cells were grown until complete sporulation observed under optical microscope. The pellet, including crystals, spores, and debris, was collected by centrifugation at 12,000*g* for 20 min at 4°C, and washed once with 1 mol/L NaCl and then with distilled water. The crystals were dissolved and digested simultaneously in 50 mmol/L Na_2_CO_3_, 3% beta‐mercaptoethanol (v/v) pH 10.0, at 37°C for 2 h in the presence of trypsin at 20:1 mass ratio (protein: trypsin). The activated toxin was purified by size‐exclusion chromatography (Superdex 75 10/300 GL, AKTA Avant, GE Healthcare, Uppsala, Sweden) using 20 mmol/L Na_2_CO_3_/NaHCO_3_ (pH 9.5) buffer at 1 mL/min flow rate. The Cry1Ac protoxin, and the purified toxin was analyzed by SDS‐PAGE (8% acrylamide), and gel was stained by Coomassie blue.

### Preparation of brush border membrane vesicles (BBMV) from *H. armigera* larvae

*Helicoverpa armigera* larvae were kindly provided by Cotton Insect Pests Laboratory (Institute of Plant Protection, Chinese Academy of Agricultural Sciences) and reared with an artificial diet (Liang et al. 1999). The 50% lethal concentration of Cry1Ac against *H. armigera* was 7.15 *μ*g/g (95% confidence interval: 2.22–17.98 *μ*g/g) as we reported previously (Xue et al. 2008). About 1000 fifth instar larvae were used for BBMV preparation. The larvae were chilled on ice for 10 min before dissection. The midgut tissue was dissected from the larvae, separated from the hindgut and fat body, a longitudinal slit was done in the tissue to remove food bolus, and it was finally washed in phosphate‐buffered saline buffer (PBS buffer: 137 mmol/L NaCl, 2.7 mmol/L KCl, 4.3 mmol/L Na_2_HPO_4_, 1.4 mmol/L KH_2_PO_4_, pH 7.4). After transfer to Eppendorf tubes, midgut tissue was quickly frozen in liquid nitrogen and stored at −70°C until use. BBMV were prepared according to the method described by Wolfersberger et al. (1987). Purified BBMV (200 *μ*g) were solubilized in 200 *μ*L 1% 3‐[(3‐cholamidopropyl)‐dimethylammonio]‐1‐propanesulfonate (CHAPS)‐containing buffer (150 mmol/L NaCl, 5 mmol/L EGTA, 20 mmol/L Tris, 1% CHAPS, w/v, pH 7.5) on ice for 30 min. After centrifugation at 13,000*g* for 5 min at 4°C, the soluble BBMV protein was quantified in using the Bradford method with bovine serum albumin (BSA) as a standard.

### Pull‐down assay

Purified Cry1Ac toxin was coupled with CNBr agarose (GE Healthcare), according to the manufacturer's instructions. One mg of purified Cry1Ac toxin was incubated with 500 *μ*L CNBr agarose in 0.2 mol/L NaHCO_3_ buffer (pH 8.3) at room temperature for 6 h. The noncoupled free CNBr was blocked by incubation with 0.1 mol/L glycine in 0.2 mol/L NaHCO_3_ buffer (pH 8.3) at room temperature for another 6 h. The noncoupled Cry1Ac protein was removed by 10 washes with 500 *μ*L PBS as described by the manufacturer. Finally, the coupled CNBr‐Cry1Ac agarose was stored in 20% ethanol (v/v) at 4°C.

We have previously described the pull‐down assay (Shu et al. 2015), which was improved to identify the Cry8Ea‐binding protein on BBMV from *Holotrichia parallela*. Briefly, after incubation of 50 *μ*L CNBr‐Cry1Ac agarose with 100 *μ*g solubilized BBMV proteins for 2 h at 4°C, the unbound BBMV proteins were removed by a fast centrifugation at 400*g* for 30 sec at 4°C. To avoid losing low abundant proteins and proteins with low affinity, the unbound proteins were recovered in the supernatant after first incubation with CNBr‐Cry1Ac and agarose, and were incubated again with additional 50 *μ*L CNBr‐Cry1Ac agarose at 4°C for 2 h. The unbound proteins were removed by centrifugation for 30 sec at 400*g* at 4°C. The CNBr‐Cry1Ac agarose containing the bound proteins was washed five times with 500 *μ*L PBS supplemented with 1 mol/L NaCl, followed by five washes with 500 *μ*L PBS to remove all unbound proteins. The proteins that remained bound to the CNBr‐Cry1Ac agarose were considered as Cry1Ac‐binding proteins and were dissociated from the agarose by boiling for 10 min in 50 *μ*L SDS‐PAGE loading buffer (100 mmol/L Tris‐Cl, 200 mmol/L DTT, 4% SDS w/v, 0.2% bromophenol blue w/v, 20% glycerol v/v, pH 6.8). The supernatant was separated by SDS‐PAGE (discontinuous acrylamide gradients from 8% to 12% acrylamide). As negative control of this experiment, the activated CNBr agarose was coupled with 0.1 mol/L Tris‐HCl amino methane buffer (pH 8.5) without Cry1Ac protein, blocked as described earlier, and used for incubation with BBMV.

### Identification of binding proteins

After stained by Coomassie blue, the SDS‐PAGE gels were divided and cut into seven parts that were sent to Huada Protein Research Center (HPRC) for LC‐MS/MS analysis. MS/MS data were searched by HPRC against ncbi_insecta_201401 201401 (1,786,564 sequences; 642,693,167 residues) database using the Mascot search engine (Matrix Science, London, UK). Search parameters are provided by HPRC as follows: type of search – MS/MS ion search; enzyme – trypsin; fixed modifications – carboxymethyl (C); variable modifications – Gln>pyro‐Glu (N‐term Q) and oxidation (M); mass values – monoisotopic; protein mass – unrestricted; peptide mass tolerance – ±15 ppm; fragment mass tolerance – ±20 mmu; max missed cleavages – 1; instrument type – default; number of queries – 9983. Peptide identifications were accepted if they could be established at greater than 50% probability as specified by the PeptideProphet algorithm (Keller et al. 2002). Protein identifications were accepted if they could be established at greater than 90% probability and contained at least two identified peptides. Protein probabilities were assigned by the ProteinProphet algorithm (Nesvizhskii et al. 2003).

## Results

### Preparation of Cry1Ac toxin

**Figure 1:**
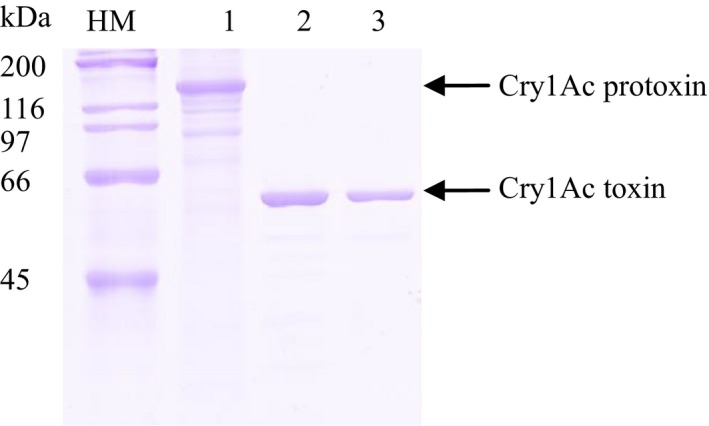
SDS‐PAGE analysis of Cry1Ac (8% acrylamide). HM: high‐range marker; 1: Cry1Ac protoxin from HD73 strain; 2: Cry1Ac toxin activated by trypsin; 3: Cry1Ac toxin purified by gel filtration chromatography.

As shown in Figure 1, Cry1Ac protoxin present in the crystal/spore mixture (lane 1) was solubilized and digested by trypsin to produce the purified activated toxin (lane 2). After purification by Superdex 75, the activated toxin (60 kDa, lane 3) was coupled with CNBr agarose as described in Experimental Procedures.

### Detection of Cry1Ac‐binding proteins in *H. armigera* BBMV

**Figure 2:**
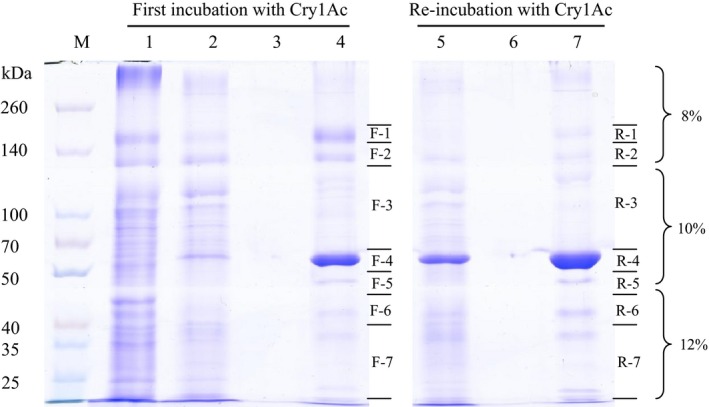
Identification of Cry1Ac‐binding proteins on the BBMV from *Helicoverpa armigera* using the pull‐down method without elution. Proteins were resolved in PAGE discontinuous acrylamide gradients from 8% to 12%. Lanes 4 and 7 were divided into seven fractions (F‐1 to F‐7 and R‐1 to R‐7) and each fraction was used for LC‐MS/MS analysis. M: marker; 1: proteins from *H. armigera* BBMV incubated with CNBr‐Cry1Ac‐coupled agarose; 2: unbound proteins after the first incubation with CNBr‐Cry1Ac agarose; 5: unbound proteins after reincubation with CNBr‐Cry1Ac agarose; 3 and 6: proteins found in the elution obtained after last wash with phosphate‐buffered saline (PBS); 4 and 7: proteins bound to CNBr‐Cry1Ac after first and reincubations, respectively.

To analyze the Cry1Ac‐binding proteins present in BBMV from *H. armigera*, BBMV were prepared from fifth instar larvae and solubilized as described in Experimental Procedures. The solubilized BBMV proteins (lane 1 in Fig. 2) were incubated with CNBr‐Cry1Ac‐coupled agarose, and the unbound proteins (lane 2 in Fig. 2) were removed by centrifugation. In order to avoid losing low‐affinity binding proteins or proteins that were less abundant, the unbound proteins (lane 2 in Fig. 2) were incubated again with CNBr‐Cry1Ac‐coupled agarose. The unbound proteins from reincubation with CNBr‐Cry1Ac agarose were removed by centrifugation (lanes 5 in Fig. 2). After exhaustive washing steps, no protein was detected in the final washing buffer according to the analysis of SDS‐PAGE electrophoresis (lanes 3 and 6 in Fig. 2). The Cry1Ac‐binding proteins in the first incubation and in the reincubation step with the CNBr‐Cry1Ac‐coupled agarose were boiled for 10 min in loading buffer and resolved in SDS‐PAGE. The gel was divided into seven regions, respectively, as shown in Figure 2 lanes 4 and 7 (F samples correspond to the first incubation step, while R samples correspond to the reincubation step). These seven regions of the gel were used for LC‐MS/MS analysis. It is important to note that the advantage of the strategy resented here is that no‐elution step was necessary to elute the insect midgut proteins bound to Cry1Ac. The bands of 60 kDa in F‐4 and R‐4 correspond to Cry1Ac toxin that was also dissociated after boiling. Protein bands in R‐1 and R‐2 became weaker compared with F‐1 and F‐2, respectively, although similar protein profiles of Cry1Ac‐binding proteins were obtained in the first incubation and in the reincubation with agarose‐coupled Cry1Ac. However, some protein bands were lost in R‐3 compared with F‐3. In the negative control performed with blocked CNBr agarose without Cry1Ac toxin, no protein bands were observed after SDS‐PAGE analysis (data not shown).

### Identification of Cry1Ac‐binding proteins by LC‐MS/MS

Table 1 shows a list of proteins identified in each fraction of the gel using a significant score higher than 50 as threshold. APN molecules were detected in all samples analyzed. Six different classes of APN as Cry1Ac‐binding proteins were identified. V‐ATPase was detected also in most samples analyzed except for F‐2, R‐1, and R‐2 samples, while polycalin was detected in most samples except for F‐5, F‐7, R‐4, and R‐5 samples. Actin was identified in F‐5 to F‐7, R‐3 and R‐5 to R‐7 samples. Alkaline phosphatase was identified in F‐3 and R‐4. Cadherin‐like protein was identified as Cry1Ac‐binding protein in F‐2 region, while ABCC2 was identified in F‐6. Also, different proteins not previously characterized as Cry1Ac‐binding proteins were identified such as dipeptidyl peptidase, carboxyl/choline esterase, azurocidin‐like serine proteinase, and trypsin‐like protease among others which had a significant score in the LC‐MS/MS analysis (Table 1).

**Table 1:** The LC‐MS/MS assay results of binding proteins to Cry1Ac on BBMVs from *Helicoverpa armigera*

Fraction	Accession number	Score	Protein description [species]^a^	Sequence coverage	Fraction	Accession number	Score	Protein description [species]	Sequence coverage
F‐1	gi|17027158	18803	Aminopeptidase N1 [Ha]	50%	R‐1	gi|30961821	7938	Midgut aminopeptidase N 2 [Ha]	54%
	gi|30961821	15683	Midgut aminopeptidase N2 [Ha]	59%		gi|17027158	1767	Aminopeptidase N1 [Ha]	42%
	gi|514430167	1367	Aminopeptidase N5 [On]	5%		gi|514430167	646	Aminopeptidase N5 [On]	4%
	gi|25814968	364	Midgut aminopeptidase APN2 [Ha]	24%		gi|30961823	434	Midgut aminopeptidase N3 [Ha]	23%
	gi|512902608	214	PREDICTED: extracellular domains containing protein CG31004‐like isoform X1 [Bm]	7%		gi|512902608	146	PREDICTED: extracellular domains containing protein CG31004‐like isoform X1[Bm]	7%
	gi|156968281	88	Multidomain lipocalin [Ha]	6%		gi|156968281	156	Multidomain lipocalin [Ha]	11%
	gi|171740913	88	Polycalin [Ha]	6%		gi|171740913	156	Polycalin [Ha]	11%
	gi|512926607	67	PREDICTED: V‐type proton ATPase 116 kDa subunit a isoform 1‐like isoform X1 [Bm]	4%		gi|27818925	156	Aminopeptidase N4 [Ha]	12%
						gi|498991524	58	PREDICTED: hydroxymethyl pyrimidine/phosphomethyl pyrimidine kinase‐like [Cc]	10%
F‐2	gi|17027158	7565	Aminopeptidase N1 [Ha]	57%	R‐2	gi|25814968	1801	Midgut aminopeptidase APN2 [Ha]	37%
	gi|30961823	3593	Midgut aminopeptidase N3 [Ha]	41%		gi|27818925	2403	Aminopeptidase N4 [Ha]	35%
	gi|30961821	4265	Midgut aminopeptidase N2 [Ha]	56%		gi|30961823	1863	Midgut aminopeptidase N3 [Ha]	40%
	gi|27818925	3902	Aminopeptidase N4 [Ha]	33%		gi|171740913	270	Polycalin [Ha]	11%
	gi|171740923	236	Polycalin [Ha]	10%		gi|294846780	91	Carboxyl/choline esterase CCE001 g [Ha]	12%
	gi|336319051	82	Dipeptidyl peptidase [Bb]	11%		gi|509177421	71	Dipeptidyl peptidase 4, partial [Pa]	23%
	gi|26051280	79	Cadherin‐like protein [Ha]	3%		gi|327420450	69	Aminopeptidase 7C [Mc]	4%
	gi|270001176	56	Hypothetical protein TcasGA2_TC016071 [Tc]	6%		gi|336319051	55	Dipeptidyl peptidase [Bb]	10%
F‐3	gi|30961821	4469	Midgut aminopeptidase N2 [Ha]	56%	R‐3	gi|11062	539	H(+)‐transporting ATPase [Ms]	27%
	gi|170791085	374	Aminopeptidase N6 [Ha]	26%		gi|30961821	448	Midgut aminopeptidase N2 [Ha]	38%
	gi|17027158	4647	Aminopeptidase N1 [Ha]	46%		gi|307695440	190	V ATPase A, partial [Ha]	42%
	gi|194295558	57	Alkaline phosphatase 2 [Ha]	18%		gi|357629674	519	V‐type proton ATPase catalytic subunit A [Dap]	28%
	gi|171740893	760	Aminopeptidase N5 [Ha]	28%		gi|156968281	409	Multidomain lipocalin [Ha]	15%
	gi|27818925	1142	Aminopeptidase N4 [Ha]	29%		gi|171740923	425	Polycalin [Ha]	15%
	gi|307695440	517	V ATPase A, partial [Ha]	62%		gi|171740893	402	Aminopeptidase N5 [Ha]	23%
	gi|171740923	1155	Polycalin [Ha]	21%		gi|27818925	399	Aminopeptidase N4 [Ha]	21%
	gi|512919199	149	PREDICTED: maltase 1‐like [Bm]	3%		gi|30961823	313	Midgut aminopeptidase N3 [Ha]	18%
	gi|30961825	1008	Midgut aminopeptidase N3 [Ha]	36%		gi|170791085	169	Aminopeptidase N6 [Ha]	20%
	gi|498991524	59	PREDICTED: hydroxymethyl pyrimidine/phosphomethyl pyrimidinekinase‐like [Cc]	10%		gi|498991524	58	PREDICTED: hydroxymethyl pyrimidine/phosphomethyl pyrimidinekinase‐like [Cc]	6%
						gi|17027158	124	aminopeptidase N1 [Ha]	17%
						gi|327420450	64	aminopeptidase 7C [Mc]	4%
						gi|15284015	60	actin [Cy]	15%
F‐4	gi|237459	1455	Vacuolar (V‐type) H(+)‐ATPase B subunit [Hv]	61%	R‐4	gi|237459	725	Vacuolar (V‐type) H(+)‐ATPase B subunit [Hv]	46%
	gi|38455217	1225	Aminopeptidase N1 [Ha]	32%		gi|25814968	118	Midgut aminopeptidase APN2 [Ha]	4%
	gi|30961823	603	Midgut aminopeptidase N3[Ha]	28%		gi|30961823	118	Midgut aminopeptidase N3 [Ha]	4%
	gi|25814968	603	Midgut aminopeptidase APN2 [Ha]	28%		gi|112820264	74	Aminopeptidase [Aj]	2%
	gi|87248463	120	ATP synthase [Bm]	17%		gi|194295558	56	Alkaline phosphatase 2 [Ha]	10%
	gi|171740913	96	Polycalin [Ha]	5%					
	gi|328709450	92	PREDICTED: hypothetical protein LOC100568862 [Ap]	5%					
	gi|456005201	53	Glycosyltransferase 1 [Cs]	4%					
F‐5	gi|17027158	639	Aminopeptidase N1 [Ha]	24%	R‐5	gi|156759	144	Actin [Dm]	25%
	gi|87248463	375	ATP synthase [Bm]	27%		gi|1419687	106	40‐kDa V‐ATPase subunit [Ms]	19%
	gi|237459	265	Vacuolar (V‐type) H(+)‐ATPase B subunit [Hv]	37%		gi|171740901	84	Azurocidin‐like serine proteinase [Ha]	26%
	gi|7158844	165	Aminopeptidase 3 [Hp]	5%		gi|87248183	84	Vacuolar ATPase subunit C [Bm]	20%
	gi|25814968	165	Midgut aminopeptidase APN2 [Ha]	12%		gi|545919627	71	Putative vacuolar H+‐ATPase V1 sector subunit c [Ca]	16%
	gi|187942442	151	Cytoplasmic actin [Ai]	47%		gi|237459	81	Vacuolar (V‐type) H(+)‐ATPase B subunit [Hv]	12%
	gi|156968281	121	Multidomain lipocalin [Ha]	9%		gi|189238960	66	PREDICTED: similar to AGAP005845‐PA [Tc]	16%
	gi|328709450	58	PREDICTED: hypothetical protein LOC100568862 [Ap]	5%					
	gi|11178	58	Glyceraldehyde‐3‐phosphate dehydrogenase [Dh]	4%					
	gi|7230426	57	Thioredoxinperoxidase 1 [Dm]	5%					
F‐6	gi|30961821	674	Midgut aminopeptidase N2 [Ha]	31%	R‐6	gi|54640128	443	ATPsyn‐beta [Drp]	36%
	gi|25814966	467	Midgut aminopeptidase APN1 [Ha]	25%		gi|509164969	255	ATP synthase [Pa]	28%
	gi|30961823	266	Midgut aminopeptidase N3 [Ha]	17%		gi|389609043	184	Vacuolar H[+]‐ATPase SFD subunit [Px]	33%
	gi|187942442	214	Cytoplasmic actin [Ai]	41%		gi|509177493	175	Vacuolar ATP synthase subunit H, partial [Pa]	31%
	gi|299481057	203	Juvenile hormone epoxide hydrolase [Ha]	20%		gi|194118083	119	GL25088 [Dp]	7%
	gi|307695438	166	V ATPase C, partial [Ha]	48%		gi|22725694	112	Aminopeptidase N [Ha]	5%
	gi|171740901	168	Azurocidin‐like serine proteinase [Ha]	45%		gi|194165198	104	GK24170 [Dw]	9%
	gi|296427826	118	ABC transporter family C protein ABCC2 [Hs]	9%		gi|156759	94	Actin [Dm]	25%
	gi|156968281	72	Multidomain lipocalin [Ha]	8%		gi|8810	86	Vacuolar ATPase B subunit [Dm]	6%
	gi|171740913	72	Polycalin [Ha]	8%		gi|171740913	85	Polycalin [Ha]	7%
	gi|328709450	52	PREDICTED: hypothetical protein LOC100568862 [Ap]	5%		gi|14269425	70	110 kDa aminopeptidase [Hv]	2%
	gi|15212555	58	Aminopeptidase N [Ha]	2%					
F‐7	gi|187942442	599	Cytoplasmic actin [Ai]	46%	R‐7	gi|187942442	576	Cytoplasmic actin [Ai]	56%
	gi|38455217	446	Aminopeptidase N1 [Ha]	30%		gi|287945	163	ATP synthase beta subunit [Dm]	28%
	gi|215434805	270	Trypsin‐like protease [Ha]	18%		gi|215434805	131	Trypsin‐like protease [Ha]	18%
	gi|25814968	255	Midgut aminopeptidase APN2 [Ha]	16%		gi|15212555	87	Aminopeptidase N [Ha]	6%
	gi|237459	234	Vacuolar (V‐type) H(+)‐ATPase B subunit [Hv]	47%		gi|389611115	85	Vacuolar H[+]‐ATPase 26kD E subunit [Pp]	9%
	gi|7158844	204	Aminopeptidase 3 [Hp]	6%		gi|403487715	74	Glyceraldehyde‐3‐phosphate dehydrogenase, partial [Cl]	22%
	gi|332025502	87	Myosin‐IB [Ae]	2%		gi|237459	70	Vacuolar (V‐type) H(+)‐ATPase B subunit [Hv]	21%
	gi|151384885	78	ADP/ATP translocase [Ha]	16%		gi|156547293	69	PREDICTED: ADP,ATP carrier protein 2‐like [Nv]	7%
	gi|40022264	76	Diazepam‐binding inhibitor [Ha]	17%		gi|194118083	65	GL25088 [Dp]	5%
	gi|18253049	60	Ribosomal protein L7 [Sf]	11%		gi|545919639	62	Putative ADP/ATP transporter on adenylatetranslocase [Ca]	10%
	gi|308055648	56	NADPH cytochrome b5 reductase [Ha]	20%		gi|171740913	55	Polycalin [Ha]	3%

Peptide score distribution. Ions score is −10log(P), where P is the probability that the observed match is a random event. Individual ions scores >38 indicate identity or extensive homology (*P* < 0.05).

a Ae, *Acromyrmex echinatior*; Ai, *Agrotis ipsilon*; Aj, *Achaea janata*; Bb, *Biston betularia*; Bm, *Bombyx mori*; Ca, *Corethrella appendiculata*; Cc, *Ceratitis capitata*; Cl, *Cymothoe lurida*; Cs, *Chilo suppressalis*; Cy, *Chironomus yoshimatsui*; Da, *Drosophila ananassae*; Dh, *Drosophila hydei*; Dm, *Drosophila melanogaster*; Dp, *Drosophila persimilis*; Drp, *Drosophila pseudoobscura*; Dw, *Drosophila willistoni*; Ha, *Helicoverpa armigera*; Hp, *Helicoverpa punctigera*; Hov, *Homalodisca vitripennis*; Hv, *Heliothis virescens*; Hs, *Heliothis subflexa*; Ms, *Manduca sexta*; Nv, *Nasonia vitripennis*; On, *Ostrinia nubilalis*; Pa, *Pararge aegeria*; Px, *Papilio xuthus*; Pp, *Papilio polytes*; Sf, *Spodoptera frugiperda*; Tc, *Tribolium castaneum*.

## Discussion

The interaction of Cry toxins and their binding proteins in the insect gut highly determines insecticidal activity. Therefore, the identification of binding proteins will facilitate the elucidation of the insecticidal mechanism of Cry toxins. Identification of binding proteins has generally been achieved using affinity chromatography (Denolf et al. 1997; Luo et al. 1997 Banks et al. 2001), ligand blot analysis (Nakanishi et al. 2002; Jurat‐Fuentes and Adang 2004; Arenas et al. 2010), or immunoprecipitation assays (Luo et al. 1996; Bravo et al. 2004). However, some binding proteins may not be identified using these methods due to denaturation of proteins or incompatibility with the elution buffer. In this article, an improved pull‐down method that does not rely on elution of binding proteins after affinity chromatography was used to identify Cry1Ac‐binding proteins on BBMV from *H. armigera*. Different Cry toxin‐receptors previously described in different insects (e.g., APN, ALP, cadherin) and several novel Cry‐interacting partners were identified, suggesting that this methodology is more comprehensive for identification of Cry toxin‐binding proteins than other methods that require protein elution. However, for many of the proteins identified here as Cry1Ac‐binding proteins, it still remains to be determined if they are involved in Cry1Ac toxicity to *H. armigera*.

Cry1Ac‐binding proteins were identified in the first incubation and reincubation with CNBr‐Cry1Ac‐coupled agarose. However, no additional Cry1Ac‐binding proteins in the reincubation step with CNBr‐Cry1Ac agarose were identified, compared with the first incubation. Moreover, ABCC2 and cadherin‐like protein, two major molecules involved in Cry1A toxin action, were only detected in the first incubation with CNBr‐Cry1Ac agarose supporting that these two proteins were not highly abundant on BBMV as was previously suggested (Zhang et al. 2012b); it is also possible that their high‐affinity binding could account for their detection only in the first incubation step. Moreover, the size of the identified protein bands corresponding to ABCC2 and cadherin‐like protein was smaller than their predicted native size, and was probably due to degradation that could also explain their identification only in the first incubation step.

Cry1Ac resistance of *H. armigera* has been shown to be linked to different cadherin allele mutations supporting that cadherin has an important role in Cry1Ac toxicity (Zhang et al. 2012a). Cadherin is well recognized as Cry toxin receptor that after binding to Cry1Ab toxins triggers toxin oligomerization, playing an important role in Cry toxin mechanism of action (Gomez et al. 2014). In the case of *H. armigera*, it was shown that residues 1217–1461 of cadherin participate in Cry1Ac interaction (Wang et al. 2005).

Among all the receptors of Cry toxins described in different insects, APN is one of the most widely studied. Binding with APN has been shown to be an important step in mediating the toxicity of Cry toxins (Bravo et al. 2013). Thirty‐eight different APN proteins have been reported for 12 different lepidopteran insects and these were classified in five groups (Pigott and Ellar 2007). In *H. armigera*, at least five different classes of APN were reported (Pigott and Ellar 2007), and APN1 and APN2 from this insect species were expressed in Hi5 insect cells showing that besides being found in the membrane and catalytically active, they were capable to bind Cry1Ac toxin (Rajagopal et al. 2003). In this article, six classes of APNs were identified as Cry1Ac‐binding proteins using the improved pull‐down method. Due to the high abundance on BBMV and degradation, many APN fragments were determined to bind with Cry1Ac, even in gel zones that are much smaller than their predicted native size. APN1 (Rajagopal et al. 2003), APN2 (Rajagopal et al. 2003), APN3 (Gill et al. 1995; Banks et al. 2001), and APN4 (Banks et al. 2001) were reported to bind Cry1Ac. In the case of APN1, it was demonstrated to be functional receptor of Cry1Ac in *H. armigera* (Sivakumar et al. 2007), and related to insect resistance to Cry1Ac (Zhang et al. 2009; Tiewsiri and Wang 2011). However, it remains to be analyzed if the other APN isoforms such as APN5 and APN6 identified here are involved in Cry1Ac toxicity in *H. armigera*. In the case of ALP, it was previously shown that ALP is involved in Cry1Ac toxicity in *H. armigera* since resistance to Cry1Ac correlated with low ALP expression in different resistant colonies (Chen et al. 2015).

Consistent with other studies, actin and V‐type ATPase A were shown to bind Cry toxins (McNall and Adang 2003; Krishnamoorthy et al. 2007; Bayyareddy et al. 2009; Chen et al. 2010). In the mosquito *Aedes aegypti*, silencing of an actin gene resulted in hypersensitive phenotype to Cry11Aa toxin suggesting that actin is somehow involved in Cry toxicity (Cancino‐Rodezno et al. 2012). Polycalin was reported to bind to Cry toxin in *B. mori* (Hossain et al. 2004; Pandian et al. 2008) and *H. armigera* (Angelucci et al. 2008; Ma et al. 2012). However, it was shown that polycalin is not likely to be involved in toxicity in *B. mori* (Pandian et al. 2010).

We identified several additional proteins with unknown function in Cry toxin mode of action such as dipeptidyl peptidase or carboxyl/choline esterase and serine proteases among others (Table 1). Further studies are required to characterize the binding interactions between Cry1Ac toxin and the proteins identified in this work and to verify the biological functions of these proteins in Cry1Ac toxicity.

The exact role of ABCC2 in the mechanism of action of Cry toxins remains elusive. Mutations in ABCC2 have been shown to be linked to high levels of Cry1Ac resistance in different lepidopteran species including *H. armigera* (Gahan et al. 2010; Baxter et al. 2011; Atsumi et al. 2012; Xiao et al. 2014). Thus, it was proposed that ABCC2 may be an additional Cry toxin receptor, but no direct evidence of binding between Cry1A toxin and ABCC2 transporter isolated from lepidopteran larvae has been provided until now. Previously, it was shown that the expression of ABCC2 from *Bombyx mori* in Sf9 insect cells provided binding of Cry1A toxins to cells and increases sensitivity to these toxins, suggesting that interaction between these proteins is important for toxicity (Tanaka et al. 2013). A previous report identified Cry1Ac‐binding proteins in *H. armigera* after a ligand blot assay performed in two‐dimensional gel electrophoresis (Chen et al. 2010). Similar proteins to those reported here were also identified such as APN, CAD, V‐ATPase, and actin (Chen et al. 2010). Some spots that showed similarities with glutathione ABC transporter from the bacteria *Erwinia carotovora* was also identified (Chen et al. 2010). However, it is important to mention that this glutathione‐ABC transporter is completely different from the ABCC2 transporter showing only 4% identity in the primary sequence with ABCC2 from *Heliothis virescence* (accession number GQ332571.1) that is linked with resistance to Cry1Ac toxin. In this work, we were able to identify an ABCC2 protein from *H. armigera* that has high similarity with an ABCC2 protein from *Heliothis subflexa* (accession number: ADH16744), which showed 96% identity with the ABCC2 protein from *H. virescens*. The identification score (−10log(P), where P is the probability that the observed match is a random event) for ABCC2 (score, 118) is in the same magnitude as cadherin (score, 79) that was previously confirmed to be a Cry1Ac‐binding protein (Wang et al. 2005). Still additional experimental evidence is required to show that Cry1Ac directly binds ABCC2 transporter. ABCC2 transporter is a transmembrane protein with a small exposed region outside the membrane (Aller et al. 2009). This structural constraint could explain why ABCC2 was not previously identified as a Cry1A‐binding protein. Pull‐down experiments allows interaction of the bait protein (Cry1Ac) with proteins in their native state. Thus, our data strongly suggests that ABCC2 is a Cry1Ac‐binding protein.

## Conclusions

APN, ALP, cadherin, actin, V‐type ATPase A, polycalin, ABCC2, and some other proteins not previously characterized as Cry toxin‐binding molecules (e.g., dipeptidyl peptidase or carboxyl/choline esterase and some serine proteases) were identified as Cry1Ac‐binding protein by the improved pull‐down method. This is the first report that provides evidence of possible direct binding of Cry1Ac toxin to ABCC2 isolated from insect BBMV.

## Conflict of Interest

None declared.
